# Epidemiology of Histologically Proven Glomerulonephritis in Africa: A Systematic Review and Meta-Analysis

**DOI:** 10.1371/journal.pone.0152203

**Published:** 2016-03-24

**Authors:** Ikechi G. Okpechi, Oluwatoyin I. Ameh, Aminu K. Bello, Pierre Ronco, Charles R. Swanepoel, Andre P. Kengne

**Affiliations:** 1 E13 Renal Unit, Groote Schuur Hospital and Division of Nephrology and Hypertension, University of Cape, Observatory, Cape Town, South Africa; 2 Department of Medicine, University of Alberta, Edmonton, Canada; 3 Institut National de la Santé et de la Recherche Médicale, Unité Mixte de Recherche Paris, France; Sorbonne Universités, Université Pierre and Marie Curie, Paris, France; and Assistance Publique-Hôpitaux de Paris, Department of Nephrology and Dialysis, Tenon Hospital, Paris, France; 4 Non-Communicable Diseases Research Unit, South African Medical Research Council & University of Cape Town, Cape Town, South Africa; Mario Negri Institute for Pharmacological Research and Azienda Ospedaliera Ospedali Riuniti di Bergamo, ITALY

## Abstract

**Background and aim:**

Glomerulonephritis (GN) is a leading cause of end-stage renal disease (ESRD) in Africa. Data on epidemiology and outcomes of glomerular diseases from Africa is still limited. We conducted a systematic review on the epidemiology of histologically proven glomerular diseases in Africa between 1980 and 2014.

**Materials and methods:**

We searched literature using PubMed, AfricaWide, the Cumulative Index to Nursing and Allied Health Literature on EBSCO Host, Scopus, African Journals online databases, and the African Index Medicus, for relevant studies. The review was conducted using standard methods and frameworks using only biopsy-confirmed data.

**Results:**

Twenty four (24) studies comprising 12,093 reported biopsies from 13 countries were included in this analysis. The median number of biopsies per study was 127.0 (50–4436), most of the studies (70.0%) originated from North Africa and the number of performed kidney biopsies varied from 5.2 to 617 biopsies/year. Nephrotic syndrome was the commonest indication of renal biopsy. The frequency of reported primary pathologic patterns included, minimal change disease (MCD); 16.5% (95%CI: 11.2–22.6), focal segmental glomerulosclerosis (FSGS); 15.9% (11.3–21.1), mesangiocapillary GN (MCGN); 11.8% (9.2–14.6), crescentic GN; 2.0% (0.9–3.5) and IgA nephropathy 2.8% (1.3–4.9). Glomerular diseases related to hepatitis B and systemic lupus erythematosus had the highest prevalence among assessed secondary diseases: 8.4% (2.0–18.4) and 7.7% (4.5–11.7) respectively. There was no evidence of publication bias and regional differences were seen mostly for secondary GNs.

**Conclusions:**

Glomerular diseases remain poorly characterized in sub-Saharan Africa due to declining renal biopsy rates and consequent paucity of data on pathologic patterns of key renal diseases. Development of renal biopsy registries in Africa is likely to enable adequate characterization of the prevalence and patterns of glomerular diseases; this could have a positive impact on chronic kidney disease evaluation and treatment in the African continent since most glomerulopathies are amenable to treatment.

## Introduction

End-stage renal disease (ESRD) is a significant public health issue worldwide, and this problem is compounded by limited access to renal replacement therapy (RRT) in developing countries [1]. Diabetes mellitus and hypertension are the major causes of ESRD in developed countries while glomerulonephritis (GN) is reported to overwhelmingly account for a high proportion of ESRD patients in developing countries [2–5]. The 2012 United States Renal data system (USRDS) shows that the adjusted ESRD incidence rates by primary cause for diabetes mellitus, hypertension and GN were 154.1/million/year, 101.1/million/year and 28.3/million/year respectively [2]. However, data from the Chinese Renal Data System show glomerular diseases as the most common cause of ESRD (57.4%) [3]. Studies from Africa have shown that glomerular diseases account for 10.2% to 52% of patients with ESRD [6–11].

The existence of well-established renal registries allows for definition and classification of the patterns of glomerular diseases in developed countries. For instance, IgA nephropathy is the commonest GN in Europe, North America and Asia [12]. With few renal registries in Africa, the patterns of renal diseases are not clearly defined due to limited data stemming from absence of relevant skills and expertise for performing renal biopsies, or appropriate tools and infrastructure to process the biopsy data when available. This creates an important gap about glomerular disease patterns in Africa despite GNs being the commonest cause of ESRD in the continent. We aimed to systematically review available published literature on the epidemiology of histologically proven glomerular diseases in Africa.

## Materials and Methods

The review was conducted using the Preferred Reporting Items for Systematic Reviews and Meta-Analyses (PRISMA) framework [13].

### Selection of eligible studies, types of studies and sources of information

Relevant studies were identified by searching PubMed, AfricaWide and the Cumulative Index to Nursing and Allied Health Literature [CINAHL) on EBSCO Host, Scopus, African Journals online (AJOL) databases, and the African Index Medicus. The search was limited to studies from African countries and to articles published between the 1^st^ of January, 1980 and 31^st^ December, 2014. The search strategy was not restricted to any language. The search was further enhanced by inspection of bibliographies of articles obtained from the databases search. Two authors (IGO and OIA) independently conducted the database searches. Abstracts from articles in French were assessed by two of the authors (OIA and APK). The reference list of articles that met the inclusion criteria were inspected for articles relevant to the review. Known regional experts in glomerular diseases were also contacted for possible additional articles that might be contained in various regional registries or unindexed local journals.

### Search Strategy

The search strategy utilized four main themes to conduct the databases search:

- Theme 1 was related to the disease of interest using Boolean operator “or” and the text-words that included “glomerulonephritis”, “glomerulonephritides”, “glomerular inflammation”, and “glomerulosclerosis”.
- Theme 2 was related to the diagnostic method for the disease of interest i.e. “renal biopsy”.
- Theme 3 was related to the population of interest using Boolean operator “or” and the text-words “child*”, “pediatric*”, “paediatric*”, “adolescents" and “adult”
- Theme 4 was related to the all countries in the region of interest using Boolean operator “or”, the text-words “Africa*”, and each individual country in the region.

Themes 1 to 4 were then combined in sequence with the Boolean operator “AND”. The search strategy within PUBMED which had returned the highest results is depicted in (S1 Table).

### Data Collection

**Fig 1:**
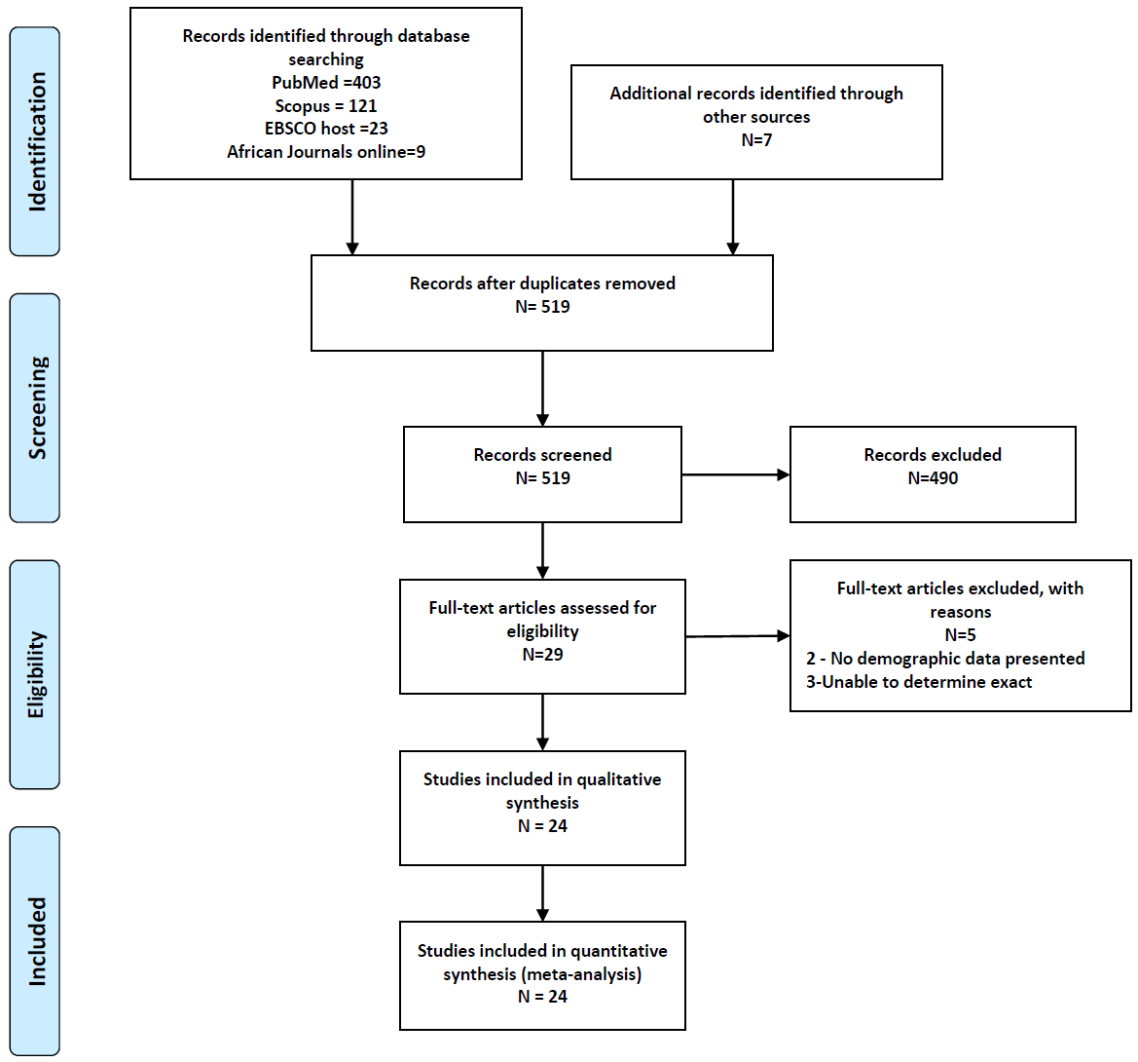
PRISMA flow diagram of study selection.

Two authors (IGO and OIA) independently assessed all abstracts for eligibility. In situations of disagreements between the two reviewers, a third reviewer (APK) arbitrated for eligibility. The inclusion criteria was that a study had to: 1) be conducted amongst African populations residing in Africa; 2) report on only biopsy-proven cases of GN; 3) enrolled at least 50 participants; 4)provided data on the demographic distribution and histopathologic types of the reported GN. We excluded studies whose focus was on: transplant related GNs, in Africans residing on other continents, entire cohort had a single or specific histopathologic type of GN, a clinical syndrome of GN without renal biopsy, and GNs reported in autopsy samples. Fig 1 shows the PRISMA study selection flowchart. A full text review of selected studies was then conducted to extract relevant data. Data collected included: year of publication, country and African region of publication, study design, number of biopsies performed, age at biopsy, gender distribution, indications for biopsy and the frequencies of reported glomerular diseases.

### Data Extraction and synthesis

The studies were categorized into 3 groups (A, B or C) depending on whether the reporting was for adults only, combined adult and paediatric study or paediatric study only, respectively. The characteristics of the selected articles extracted include the main indication for renal biopsy, age of patients at the time of biopsy, the period over which the biopsies had been done, and the number of biopsies performed in the period. The average number of biopsies performed per study was calculated as the total number of biopsies over the entire period divided by the total duration of the study. The frequencies of primary and secondary GNs were determined. Secondary GNs selected for reporting were limited to lupus nephritis (LN), GNs related to human immunodeficiency virus (HIV) and hepatitis B viral infections and amyloidosis as these were the commonly reported forms of secondary GNs. Data extraction was done by one author (IGO) and another author (OIA) verified the accuracy and validity of extracted data. To generate the frequency of different GNs across studies, the DerSimonian-Laird random effects models were used to pool study level estimates overall and by geographic region [North Africa, East and Central Africa, Southern Africa, and West Africa]. To minimise the effect of extreme proportions on the overall estimates, we first stabilized the variance of the raw prevalence with a single arcsine transformation before pooling [14]. Heterogeneity across studies was assessed with the use of the Cochran Q and I^2^ statistics [15], while the Egger test of bias [16] was used to investigate the publication bias. All analyses used the R statistical software (version 3.0.3 [2014-03-06], The R Foundation for statistical computing, Vienna, Austria) and ‘*meta*’ package (version 4.1–0).

## Results

### Demographic and clinical features

Twenty-four full papers were reviewed and analysed for this study [17–40]. Eight of the 24 studies (33.3%) reported on studies in adults (group A) [17–24], 11/24 (45.8%) comprised adults and paediatrics (group B) [25–35] and 5/24 studies (20.8%) were restricted to paediatric age groups (group C) [36–40]. Overall, 12,093 renal biopsies were reported in the 35 year period evaluated. The period of analysis ranged from as short as 12 months to up to 30 years and only 3 studies were found to have had a prospective design; no study was reported from a national renal biopsy registry. Most of the studies in all the groups had a male predominance: 45.2%–62.7% for studies in group A; 47.3%–64.3% for studies in group B and 53.0%–68.8% for studies in group C. The average age at time of biopsy for the studies in group A was less than 40 years, while group C studies had an average age less than 9.5 years. Overall, the median number of biopsies per study was 127.0 (50–4436), however, the rate of renal biopsies reported per year showed extensive variation (group A: 17.1/year to 134/year; group B: 7.9/year to 617/year and group C: 5.2/year to 83.1/year). Nephrotic syndrome was the most common indication for renal biopsy and in 11/24 studies (45.8%), nephrotic syndrome was the sole reason (100%) for performing a biopsy [18–20, 25–29, 34, 36–38] (Table 1). Overall, 11/24 studies (45.8%) were published from the year 2000 onwards with 8 from North Africa, 2 from West Africa and 1 from Southern Africa [21–24, 31–35, 39, 40]. Eight studies did not use the special stains (immunofluorescence or immunohistochemistry or immunoperoxidase) for evaluation of renal histology [17, 19, 20, 25, 26, 28, 29, 35]. Two studies reported that the special stains were performed in Europe (UK and France) [30, 32].

**Table 1:** Demographic features of patients in included studies.

Year of publication	Region	Country	Author [REF]	Study period (duration)	Study design	Number of biopsies reported	Average age at biopsy (years)	Males (%)	Biopsy rate (number/year)	Main indication for renal biopsy (%)
	**Group A: Studies reporting adult biopsies only**									
1984	West Africa	Nigeria	Awunor-Renner et al [17]	NR (1 year)	NR	134	NR (12–61)	55.2	134.0	NS (50)
1992	West Africa	Nigeria	Oviasu et al [18]	1982–1988 (7 years)	NR	120	Males: 37.5 (19–60), Females: 29.6 (15–52)	56.7	17.1	NS (100)
1993	East and Central Africa	Zaire	Pakasa et al [19]	1986–1989 (4 years)	NR	92	30.9 ± 13.2	48.0	23.0	NS (100)
1993	West Africa	Nigeria	Ojogwu et al [20]	NR	NR	88	28.9 ± 8.6	60.2	NR	NS (100)
2008	West Africa	Senegal	Niang et al [21]	1993–2003 (11 years)	Retrospective	258	28 (15–79)*	NR	25.9	NS (39.8)
2011	Southern Africa	South Africa	Okpechi et al [22]	2000–2009 (10 years)	Retrospective	1,284	36.8 ± 14.0	45.2	128	NS (52.5)
2012	North Africa	Morocco	Aatif et al [23]	2000–2007 (8 years)	Retrospective	161	40.4 ± 15.0	62.7	20.1	NS (60.3)
2013	North Africa	Sudan	Nadium et al [24]	2010–2011 (1 year)	Prospective	71	34.6 ± 18	54.9	71.0	NS (46.5)
	**Group B: Studies with combined adult and paediatric biopsies**									
1980	East and Central Africa	Kenya	Kung’u et al [25]	1970–1978 (9 years)	NR	150	(1 –>60)	50.7	16.7	NR
1981	West Africa	Ghana	Adu et al [26]	NR (4 years)	Retrospective	61	Children: 7 (3–11), Adults: 26 (13–50)	59.0	15.3	NS (100)
1990	East and Central Africa	Cameroon	Mbakop et al [27]	1986–1989 (4 years)	Retrospective	50	(8–53)	NR	12.5	NS (100)
1993	West Africa	Nigeria	Kadiri et al [28]	1985–1990 (4.5 years)	NR	84	10–59	64.3	18.7	NS (100)
1994	East and Central Africa	Kenya	McLigeyo SO [29]	1982–1993 (12 years)	NR	422	28.4 ± 9.2	49.5	35.2	NS (100)
1997	Southern Africa	Zimbabwe(#)	Borok et al [30]	1982–1988 (6 years)	Prospective	119	(3–52)	55.0	19.8	NS (64.0)
2000	North Africa	Egypt	Barsoum et al [31]	1998–1999 (2 years)	Retrospective	1,234	30.5 ± 17.4	54.1	617	NS (31.8)
2001	West Africa	Senegal	Diouf et al [32]	1993–1998 (6 years)	Retrospective	115	28 (5–60)	55.7	19.2	NS (67.0)
2006	North Africa	Tunisia	Ben Maiz et al [33]	1975–2005 (30 years)	Retrospective	4,436	(≤15–≥65)	NR	147.9	NR
2011	North Africa	Morocco	Ayach et al [34]	2000–2009 (9.75 years)	Retrospective	77	(12–25)	61.0	7.9	NS (100)
2012	North Africa	Egypt	Ibrahim et al [35]	2003–2008 (5 years)	Retrospective	924	26.5 ± 14.6	47.3	184.8	Proteinuria (43.1)
	**Group C: Studies reporting paediatric biopsies only**									
1990	West Africa	Nigeria	Abdurrahman et al [36]	NR (4years)	Prospective	98	5.8 (1–12yrs)	53.0	24.5	NS (100)
1997	Southern Africa	South Africa	Bhimma et al [37]	NR (20 years)	Retrospective	480	5.7 (2weeks–16 years)	68.8	24.0	NS (100)
1999	Southern Africa	Namibia	Van Buuren et al [38]	1975–1988 (13 years)	Retrospective	68	7.2 (1.8–15)	68.6	5.2	NS (100)
2010	North Africa	Sudan	Abdelraheem et al [39]	2002–2007 (6 years)	Retrospective	321	8.7 (2 months–16 years)	60.2	53.5	NS (62.9)
2014	North Africa	Egypt	Bakr et al [40]	1998–2012 (15 years)	Retrospective	1246	9.2 ± 3.7	53.9	83.1	NS (48.5)

NR–Not reported; NS–Nephrotic syndrome; (#)–Biopsies were performed in Zimbabwe but the specimens were processed at St Mary’s Hospital, UK

* Median age (minimum–maximum).

### Distribution of glomerular disease patterns

Table 2 shows regional differences of types of GN as reported from the various studies. The proportion of biopsies performed for the search period per region was 70.0%, 16.1%, 7.9% and 5.9% for North Africa, Southern Africa, West Africa and East and Central Africa respectively. With the exception of diffuse proliferative GN (DPGN) (p = 0.0005), the contribution of primary aetiologies to the distribution of GNs across regions was mostly similar (all p≥0.099 for regional differences). With regard to secondary GNs, significant regional differences were apparent (all p<0.052), although some comparisons were based on very few outcomes. For both primary and secondary GNs, there was indication of significant within region heterogeneities for some of the outcomes. But within region heterogeneities have to be interpreted against the background of small number of eligible studies within regions.

**Table 2:** Frequencies of glomerular diseases by African regions.

GN Type	Regions	N studies	N biopsies	N outcomes	% (95% CI)	I^2^	P heterogeneity	p-diff Regions
**MCD**	ECAf	4	714	139	20.6 (15.0–26.8)	66.3	0.031	0.406
	NAf	7	4034	612	22.5 (12.6–34.3)	98.4	<0.0001	
	SAf	4	1951	211	11.2 (0.3–34.5)	99.1	<0.0001	
	WAf	8	958	127	12.2 (4.4–23.1)	95.0	<0.0001	
**MesPGN**	ECAf	2	472	109	14.7 (1.5–38.1)	92.7	0.0002	0.771
	NAf	5	2711	249	7.0 (4.0–10.8)	89.2	<0.0001	
	SAf	3	1832	127	8.9 (4.9–14.1)	87	0.0005	
	WAf	3	287	29	9.5 (1.5–23.3)	90.6	<0.0001	
**MCGN**	ECAf	4	714	65	9.1 (7.1–11.3)	0	0.822	0.087
	NAf	7	8393	785	9.7 (6.6–13.4)	95.1	<0.0001	
	SAf	4	1951	130	6.4 (3.8–9.6)	75.8	0.006	
	WAf	7	843	161	20.7 (8.9–35.7)	95.8	<0.0001	
**FSGS**	ECAf	4	714	118	15.5 (5.7–29.0)	93.4	<0.0001	0.889
	NAf	7	4034	543	13.2 (9.5–17.5)	91.4	<0.0001	
	SAf	4	1951	212	14.5 (2.8–33.2)	98.5	<0.0001	
	WAf	8	958	265	19.1 (5.9–37.4)	97.6	<0.0001	
**MGN**	ECAf	4	714	74	12.5 (7.1–19.2)	78.3	0.0032	0.142
	NAf	8	8470	649	5.7 (2.2–10.5)	98.1	<0.0001	
	SAf	4	1951	127	5.7 (2.1–9.9)	89.4	<0.0001	
	WAf	8	958	70	5.9 (3.0–9.6)	78.1	<0.0001	
**IgAN**	ECAf	1	50	0	0.0 (0.0–1.9)	-	-	0.099
	NAf	6	7469	332	3.8 (1.6–6.9)	96	<0.0001	
	SAf	1	1284	26	2.0 (1.3–2.9)	-	-	
	WAf	1	115	2	1.7 (0.2–4.9)	-	-	
**PIGN**	ECAf	1	50	0	0.0 (0.0–1.9)	-	-	0.102
	NAf	2	482	81	10.3 (0.0–41.3)	98.4	<0.0001	
	SAf	2	1352	38	2.8 (2.0–3.7)	0	0.431	
	WAf	1	115	3	2.6 (0.5–6.3)	-	-	
**CresGN**	ECAf	3	622	41	3.9 (0.6–10.0)	85.2	0.0012	0.565
	NAf	3	2641	32	1.4 (0.5–27.4)	80.2	0.0064	
	SAf	2	1403	52	2.4 (0.2–6.6)	80.6	0.023	
	WAf	4	467	7	1.1 (0.05–3.4)	68.7	0.023	
**DPGN**	ECAf	4	714	82	10.3 (4.1–18.8)	88	<0.0001	0.0005
	NAf	5	8001	602	4.6 (1.1–10.2)	98.7	<0.0001	
	SAf	1	119	28	23.5 (16.4–31.5)	-	-	
	WAf	5	497	98	17.8 (9.1-28-7)	88.2	<0.0001	
**FPGN**	ECAf	1	422	6	1.4 (0.5–2.8)	-	-	0.134
	NAf	3	3404	251	6.5 (2.1–13.2)	97.8	<0.0001	
	SAf	1	119	4	3.4 (0.9–7.3)	-	-	
	WAf	4	399	13	2.3 (0.1–7.2)	82.9	0.0006	
**Lupus Nephritis**	ECAf	1	422	6	1.4 (0.5–2.8)	-	-	<0.0001
	NAf	6	8322	1122	13.9 (8.9–19.9)	97.6	<0.0001	
	SAf	3	1471	254	6.1 (0.0–22.5)	96.8	<0.0001	
	WAf	4	554	19	3.0 (0.1–9.8)	90.6	<0.0001	
**Hep B**	ECAf	1	422	0	0.0 (0.0–0.2)	-	-	<0.0001
	NAf	1	321	10	3.1 (1.5–5.3)	-	-	
	SAf	3	1832	116	14.5 (0.7–41.2)	99.1	<0.0001	
	WAf	3	352	53	11.0 (0.0–39.4)	97.6	<0.0001	
**HIVAN**	ECAf	None	-	-	-	-	-	0.233
	NAf	1	321	0	0.0 (0.0–0.3	-	-	
	SAf	2	1764	145	2.9 (0.0–23.6)	99.4	<0.0001	
	WAf	1	258	1	0.4 (0.0–1.5)	-	-	
**Amyloid**	ECAf	3	664	15	3.0 (0.2–6.7)	88	0.0002	0.052
	NAf	6	8078	559	4.3 (1.4–8.6)	97.9	<0.0001	
	SAf	2	1403	16	1.1 (0.6–1.8)	0	0.596	
	WAf	6	772	18	2.2 (1.3–3.4)	0	0.638	

MCD–Minimal change disease; MesPGN–Mesangial proliferative GN, MCGN–Mesangiocapillary GN; FSGS–Focal segmental glomerulosclerosis; MGN–Membranous GN; IgAN–Ig A nephropathy; PIGN–Post-infectious GN; CresGN–Crescentic GN; DPGN–Diffuse proliferative GN; FPGN–Focal proliferative GN; Hep. B–Hepatitis B; HIVAN–Human immunodeficiency virus associated nephropathy, WAf–West Africa, ECAf–East and Central Africa, SAf–Southern Africa, NAf–North Africa.

**Fig 2:**
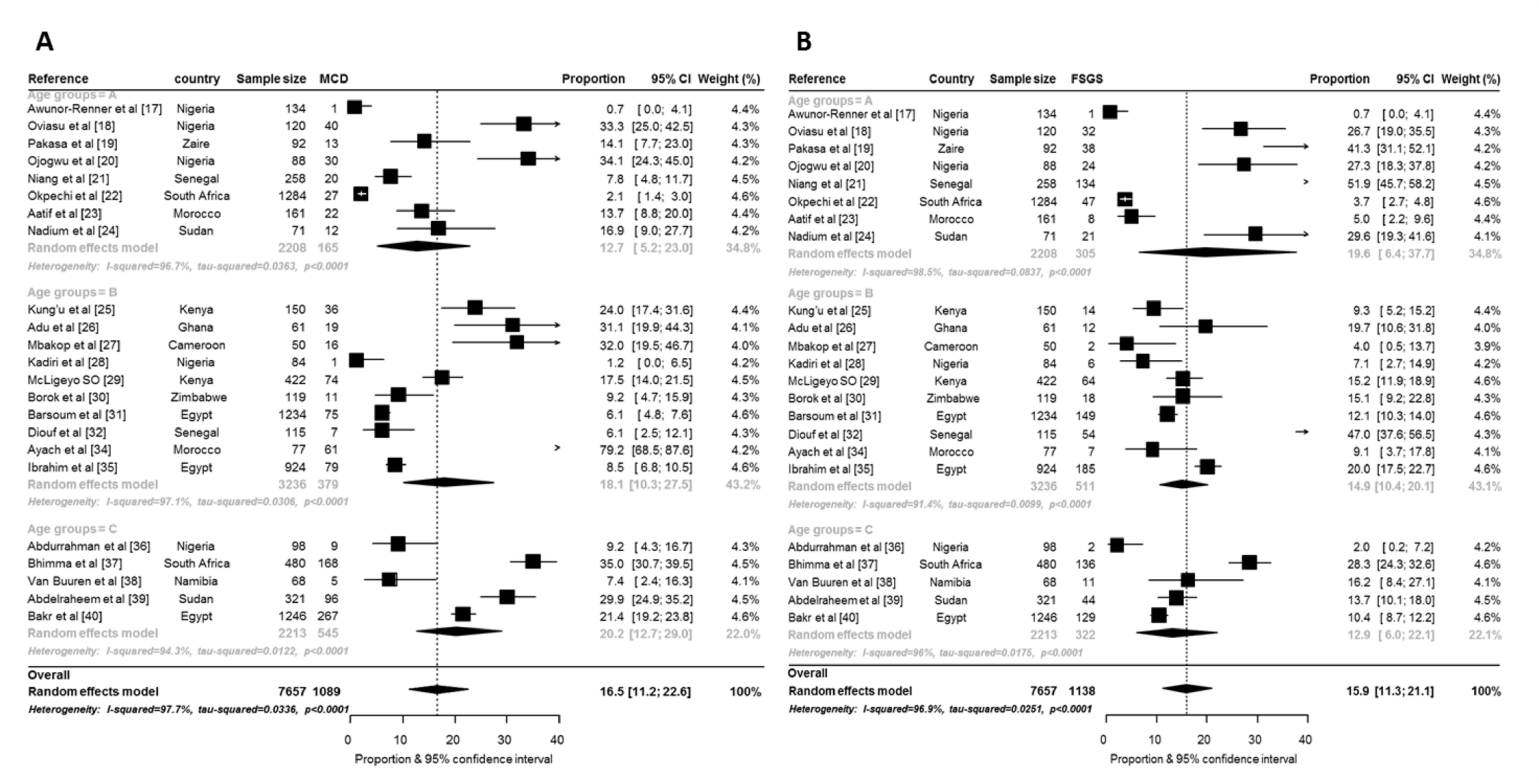
Forest plot for random-effects meta-analyses for MCD and FSGS. This figure shows the pooled prevalence of minimal change disease (2A) and focal segmental glomerulosclerosis (2B) overall and by age group (MCD–minimal change disease, FSGS–focal segmental glomerulosclerosis).

**Fig 3:**
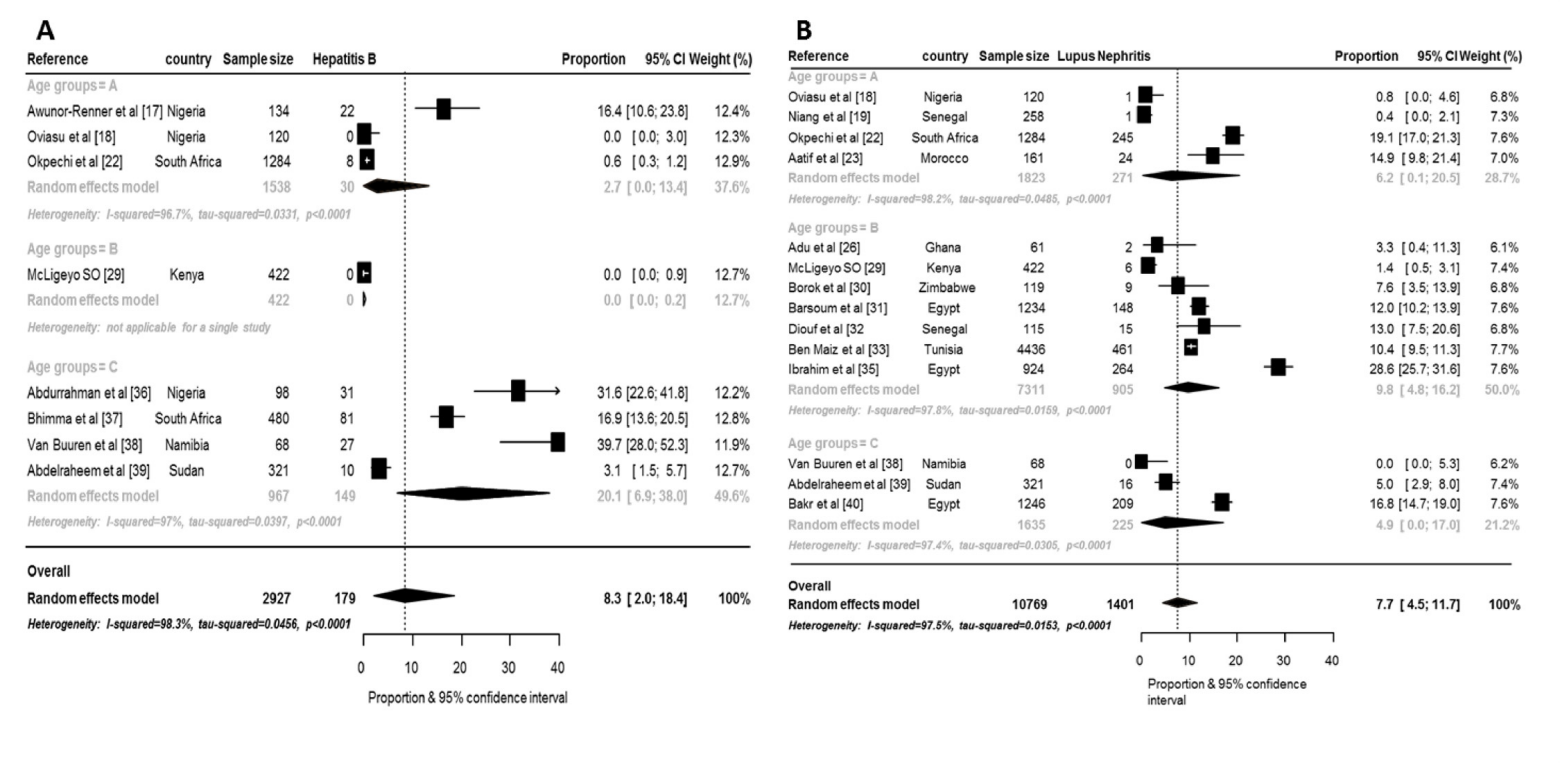
Forest plot for random-effects meta-analyses for hepatitis B related GNs and lupus nephritis. This figure shows the pooled prevalence of hepatitis B related GNs (3A) and lupus nephritis (3B) overall and by age group.

The prevalence of the various GNs according to countries and age at time of diagnosis is summarized in Figs 2 and 3 and in S1–S5 Figs. The overall prevalence of minimal change disease (MCD) was the highest of all GNs reported by all 24 studies at 16.5% (95%CI: 11.2–22.6; n = 7657; p-heterogeneity <0.0001). The highest prevalence of MCD was from a group B study in Morocco: 79.2% (68.5–87.6) [34] while 2 studies from Nigeria reported the lowest prevalence [17, 28]. Paediatric studies (group C) had the highest prevalence of MCD (compared to group A and B) but there was no significant difference across age groups (p = 0.496) (Fig 2A). There was no significant regional difference in overall prevalence of MCD in Africa (p = 0.406) although North Africa and East and Central African countries had the highest prevalence of MCD (Table 2).

The overall prevalence of focal segmental glomerulosclerosis (FSGS) pattern was 15.9% (11.3–21.1; p-heterogeneity <0.0001) (Fig 2B). Two studies from Senegal reported the highest prevalence rates: 51.9% (45.7–58.2) [21] and 47.0 (37.6–56.5) [32]. Awunnor-Renner et al [17] reported the lowest prevalence from adults in Zaria, Nigeria: 0.7% (0.0–4.1) while the lowest prevalence in paediatric studies was also reported from another study from Zaria, Nigeria [36]. However, other studies from Nigeria reported prevalence rates as high as 27.3% [20]. The prevalence of FSGS according to regions was highest for West Africa: 19.1% (5.9–37.4) and lowest for North Africa: 13.2% (9.5–17.5) but without significant interregional differences (p = 0.889) (Table 2).

The prevalence of other reported primary GN patterns were: 9.2% (95%CI: 6.2–12.7; p-heterogeneity <0.0001, difference across age groups: p = 0.511) for mesangial proliferative GN (MesPGN), 11.8% (95%CI: 9.2–14.6; p-heterogeneity <0.0001, difference across age groups: p = 0.435) for mesangiocapillary GN (MCGN), 6.6% (95%CI: 4.6–9.0; p-heterogeneity <0.0001, difference across age groups: p = 0.0001) for membranous GN (MGN), 2.8% (95%CI: 1.3–4.9; p-heterogeneity <0.0001, differences across age groups: p = 0.0039) for IgA nephropathy (IgAN), 3.5% (95%CI: 0.2–10.9; p-heterogeneity <0.0001, differences across age groups: p = 0.467) for post-infectious GN (PIGN), 2.0% (95%CI: 0.9–3.5; p-heterogeneity <0.0001, differences across age groups: p = 0.862) for crescentic GN, 10.7% (95%CI: 6.8–15.3; p-heterogeneity <0.0001, differences across age groups: p = 0.096) for DPGN and 3.6% (95%CI: 1.4–6.8; p-heterogeneity <0.0001, differences across age groups: p<0.0001) for focal proliferative GN (FPGN). Only 4 primary disease patterns (MCD, MCGN, FSGS and MGN) were reported from more than 20 of the 24 studies. There was no evidence of publication bias across all studies on primary GNs, all p≥0.143 for the Egger test of bias (S1–S4 Figs).

**Fig 4:**
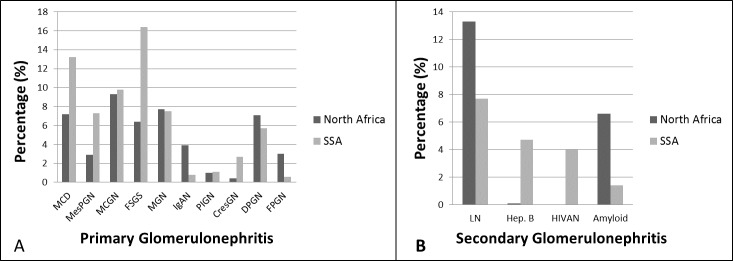
Regional differences (North Africa vs sub-Saharan Africa) in the prevalence of GNs. This figure shows the regional differences in the prevalence of primary GNs (4A) and secondary GNs (4B) for North Africa and sub-Saharan Africa.

Glomerular diseases related to hepatitis B virus infection had the highest prevalence among the reported secondary GNs: 8.4% (95% CI: 2.0–18.4; p-heterogeneity <0.0001, differences across age groups: p<0.0001) (Fig 3A). Lupus nephritis (LN) had the second highest prevalence: 7.7% (95%CI: 4.5–11.7; p-heterogeneity <0.0001, differences across age groups: p<0.0001) (Fig 3B). The high prevalence of LN was due to the high prevalence from studies from North Africa (p for difference across regions <0.0001). Surprisingly, the human immunodeficiency virus associated nephropathy (HIVAN) had an overall prevalence of 1.0% (95%: 0.0–9.0; p-heterogeneity <0.0001, differences across age groups: p<0.0001). Amyloid was present in 2.9%. There was no evidence of publication bias, all p≥0.082 for the Egger test of bias (Fig 3, S5A and S5B Fig). Fig 4A and 4B shows the crude prevalence of primary and secondary GNs in North Africa and sub-Saharan Africa (SSA) with MCGN as the commonest primary GN in North Africa (9.3%) while FSGS is the most common in SSA (16.4%). Of the secondary GNs, LN was more common in North Africa while all reported HIVAN was from SSA.

## Discussion

There is paucity of reliable data on the epidemiology of ESRD in many parts of Africa partly due to the unavailability of renal registries. In Africa, glomerular diseases have been reported to account for a large proportion of patients with ESRD from several studies (reviewed in Maoujoud et al) [5]; in many of such studies, the diagnosis of GN may have been assumed given that late presentation of patients is common in Africa. In this review, we have found that: (i) most renal biopsies reported from Africa originates from studies in North Africa, (ii) MCD, FSGS and MCGN are the most common patterns of glomerular diseases reported in Africa, (iii) Hepatitis B and LN are common and important secondary causes of GNs and (iv) lack of standard and consistency in reporting renal biopsies.

Of the 12,093 biopsies that were reported, 70% were from studies carried out in North Africa and these countries had more papers published after the year 2000 compared to countries from sub-Sahara Africa. Due to cost (out-of-pocket payment), absence of skills for performing biopsies, handling of tissue and adequate interpretation of histology few centres in Africa are able to regularly perform renal biopsies (IGO, one of the co-authors can personally confirm this from meetings as an ISN educational ambassador in Africa).

Overall, MCD, FSGS and MCGN patterns of glomerular diseases were the most frequently reported GNs. This may be because in 11 of the 24 studies, nephrotic syndrome was the sole indication for performing a renal biopsy [18–20, 26–29, 34, 36–38] and these conditions are common causes of nephrotic syndrome. We observed that only six studies [17, 22–24, 34, 40] differentiated the glomerular disease patterns into primary and secondary causes. Hence, the proportion of the reported diagnoses/patterns that were truly of primary cause is unclear. For instance, although a number of studies in this review showed that MCGN is common, recent reports [41] have proven the rarity of true primary MCGN if appropriate diagnostic algorithms were applied. However, one study from South Africa reported primary MCGN to be common after an exhaustive search for secondary causes and also reported poor prognostic factors associated with MCGN [42]. We think that the high prevalence of FSGS may be related to a limited evaluation for secondary causes. A key study by Van Rensburg et al from Bloemfontein South Africa found FSGS to be the most common histological diagnosis in a 10 year review of medical renal diseases [43], but they reported that all FSGS (including HIV) were grouped together. The role of genetic factors such as the APOL-1 which have been shown to be strongly associated with FSGS (either primary or secondary association with HIV) in Africans [44, 45] supports our observation of an increased prevalence of FSGS in Africa.

Sub-Sahara Africa has the highest prevalence of hepatitis B virus infection in comparison to other regions in the world [46], hence this may account for the high prevalence of GNs related to hepatitis B infection. Glomerular diseases associated with hepatitis B were common in children from West and Southern Africa [36–38]. We speculate that the observed prevalence may be much lower than true prevalence due to under-reporting of cases. However, the studies [17, 18, 22, 29, 36–39] that reported GNs in hepatitis B positive patients did not show direct evidence for viral particles in the glomerulus. The result of the meta-analysis done for secondary GNs is perhaps related to the low performance and reporting of renal biopsies. Further, sub-Saharan Africa still bears an inordinate share of the global HIV burden. In 2014, that number reached 25.8 million [24.0 million –28.7 million], 70% of the global burden of HIV [47]. Given the very high HIV burden in SSA, HIVAN would be expected to be the secondary GN with the highest prevalence. However, this was not the case partly due to underperformance and reporting of renal biopsies across Africa. We have previously reported a rising incidence in biopsies associated with HIV in Cape Town from 6.6% in 2000 to 25.7% in 2009 [22] and this trend may apply to other countries with a high burden of HIV. Also, many studies from Africa describe renal disease in HIV positive patients without performing a renal biopsy. For example, Emem et al from Nigeria described the prevalence, clinical features and risk factors of renal disease in 400 consecutive HIV/AIDS patients presenting to their centre. However, only 10 patients had a renal biopsy out of which 7 (70%) had FSGS and the histology in others were reported as normal [48].

Similarly, LN is increasingly diagnosed in Africans especially from South Africa and countries of North Africa. The study from Tunisia clearly showed a changing pattern in the epidemiology GN over time and suggested that improving socio-economic factors might have played a role in the increasing prevalence of LN and IgA nephropathy while conditions often associated with chronic infections (e.g. MCGN and Amyloidosis) had reduced within three decades [33]. A disease entity we did not find reported during the assessed period was “malaria nephropathy” which used to be commonly reported from studies in the 60s and 70s [49, 50]. Whether this is related to improving socio-demographic factors or better and clearer diagnostic methods is not known. However, a number of authors have recently disputed the evidence for malarial nephropathy [51, 52].

We acknowledge key limitations of this work including lack of immunofluorescence technology in most of the studies and the absence of uniformity in describing pathologic patterns. For instance, most studies published before the year 2000 often referred to diffuse proliferative and focal proliferative GNs without further elaboration of the pattern (e.g. crescentic, necrotizing lesions or vasculitis). As many of the studies did not say if further serological, immunohistochemistry or immunofluorescence tests were conducted, some of the reported cases of DPGN / FPGN may have fallen under categories such as crescentic GN or PIGN.

Further, the small number of studies and studies with limited samples precluded careful investigation of the substantial heterogeneities observed across studies by subgroup and meta-regression analyses. It is known, however that statistical approaches to assessing heterogeneity could yield spurious results within uncontrolled studies. Hence, given that many of the studies included in this review were inherently weak, caution must be exercised in the interpretation of the results especially with regard to the prevalence of HIVAN as observed and reported. Although this is a weakness of this study, we are however confident that the results present a perspective of GN types commonly encountered in Africa. Although the performance of renal biopsies is necessary for diagnosis of GNs, we also acknowledge that this is not feasible in most African countries where the facilities and expertise are limited. There is therefore an urgent need to support initiatives (such as the ISN’s educational ambassador program) to improve the expertise in renal biopsy performance, tissue processing and interpretation especially in SSA where this skill is typically absent. By doing this, renal biopsy registries are likely to become developed in Africa. This is not impossible despite the numerous challenges and will enhance our understanding of the epidemiology of GNs in Africa.

In conclusion, GNs in Africa are poorly characterized, especially in sub-Saharan Africa where the number of diagnostic biopsies performed has continued to decline over the last decade. Understanding the epidemiology of GNs in Africa is vital for an optimal care of patients with such conditions that are frequently associated with ESRD in this region. This could impact positively on CKD management in Africa as GNs are amenable to treatment in instances where they are diagnosed early enough. There is need to develop initiatives that assist in improving the expertise in renal biopsy performance, tissue processing and interpretation in Africa.

## Supporting Information

S1 PRISMA Checklist PRISMA Checklist. (DOC)

S1 Fig Forest plot for random-effects meta-analyses for MCGN and MesPGN. This figure shows the pooled prevalence of mesangiocapillary GN [MCGN] (1A) and mesangial proliferative GN [MesPGN] (1B) overall and by age group.
(PPTX)

S2 Fig Forest plot for random-effects meta-analyses for MGN and IgAN. This figure shows the pooled prevalence of membranous GN [MGN] (2A) and IgA nephropathy [IgAN] (2B) overall and by age group.
(PPTX)

S3 Fig Forest plot for random-effects meta-analyses for crescentic GN and PIGN. This figure shows the pooled prevalence of crescentic GN (3A) and post-infectious GN [PIGN] (3B) overall and by age group.
(PPTX)

S4 Fig Forest plot for random-effects meta-analyses for DPGN and FPGN. This figure shows the pooled prevalence of diffuse proliferative GN [DPGN] (4A) and focal proliferative GN [FPGN] (4B) overall and by age group.
(PPTX)

S5 Fig Forest plot for random-effects meta-analyses for HIVAN and Amyloidosis. This figure shows the pooled prevalence of HIV associated nephropathy [HIVAN] (5A) and amyloidosis (5B) overall and by age group.
(PPTX)

S1 Table PUBMED search strategy. (DOCX)
