# Supplementary figures and images for: Epidemiology of Histologically Proven Glomerulonephritis in Africa: A Systematic Review and Meta-Analysis
Source: PLoS One. 2016 Mar 24;11(3):e0152203. doi: 10.1371/journal.pone.0152203 (PMC4806979; doi:10.1371/journal.pone.0152203)

## Slide 1
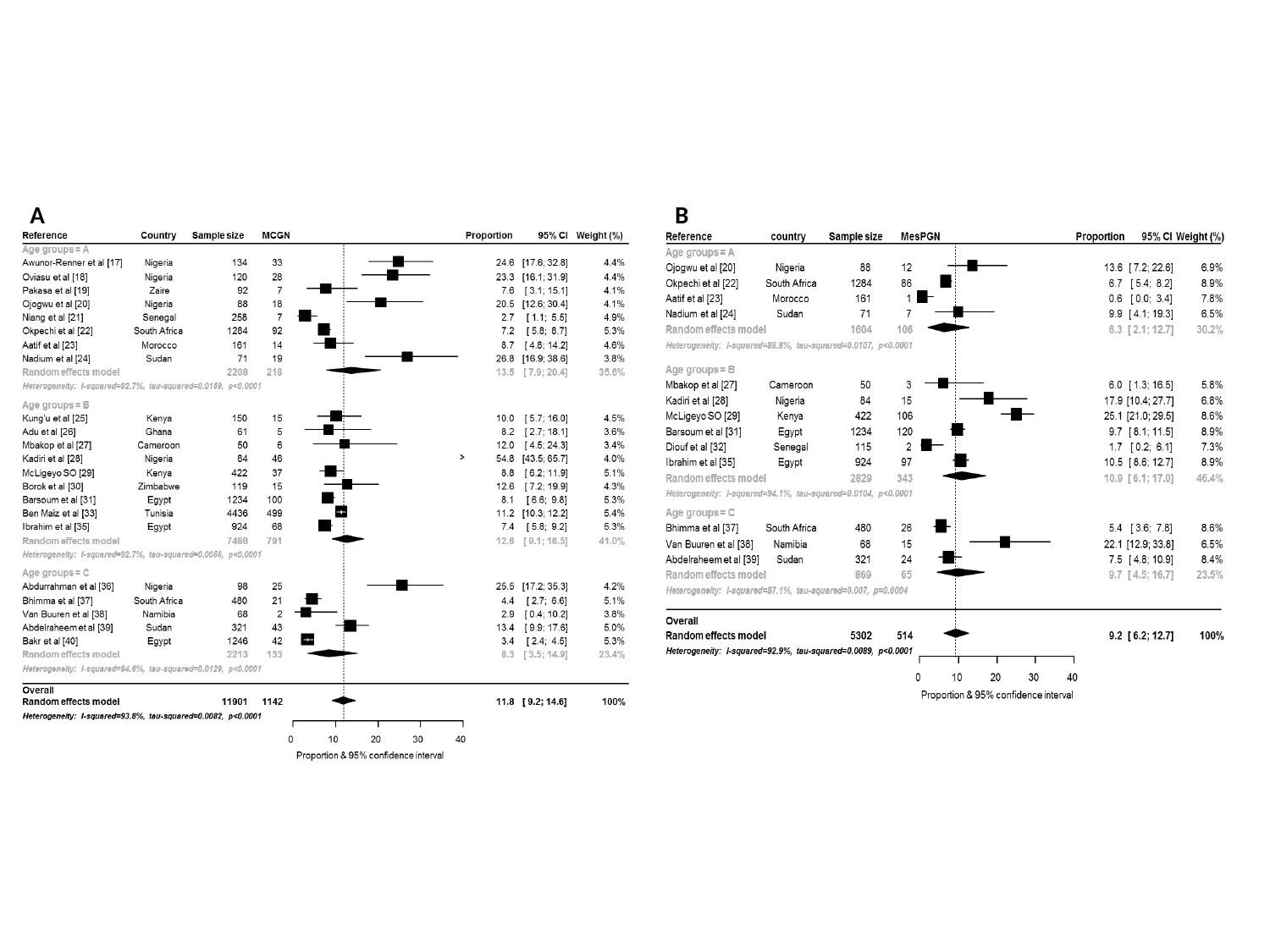

A
B

Supplement: S1 Fig — This figure shows the pooled prevalence of mesangiocapillary GN [MCGN] (1A) and mesangial proliferative GN [MesPGN] (1B) overall and by age group. (PPTX) [file pone.0152203.s002.pptx]

## Slide 1
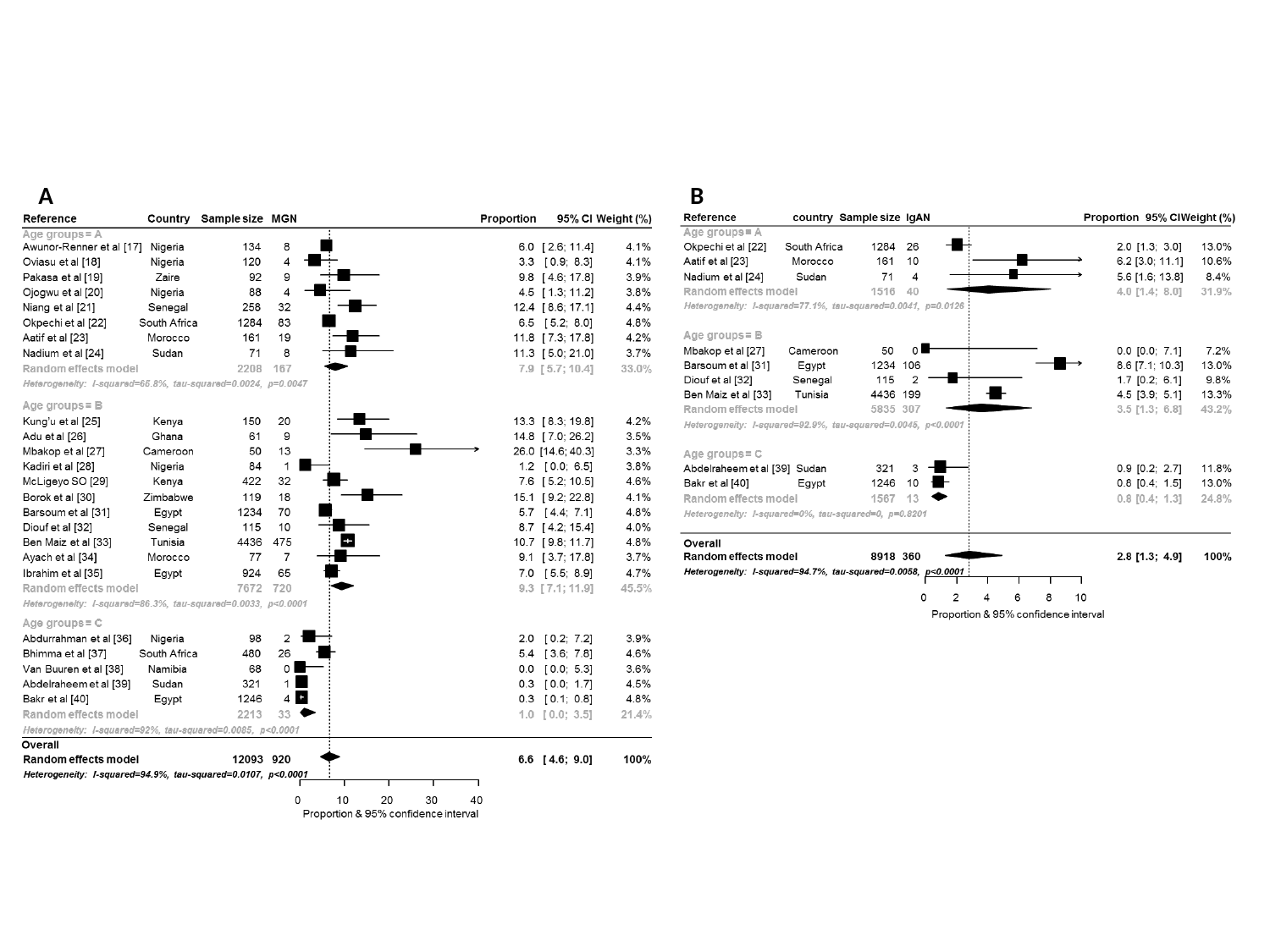

A
B

Supplement: S2 Fig — This figure shows the pooled prevalence of membranous GN [MGN] (2A) and IgA nephropathy [IgAN] (2B) overall and by age group. (PPTX) [file pone.0152203.s003.pptx]

## Slide 1
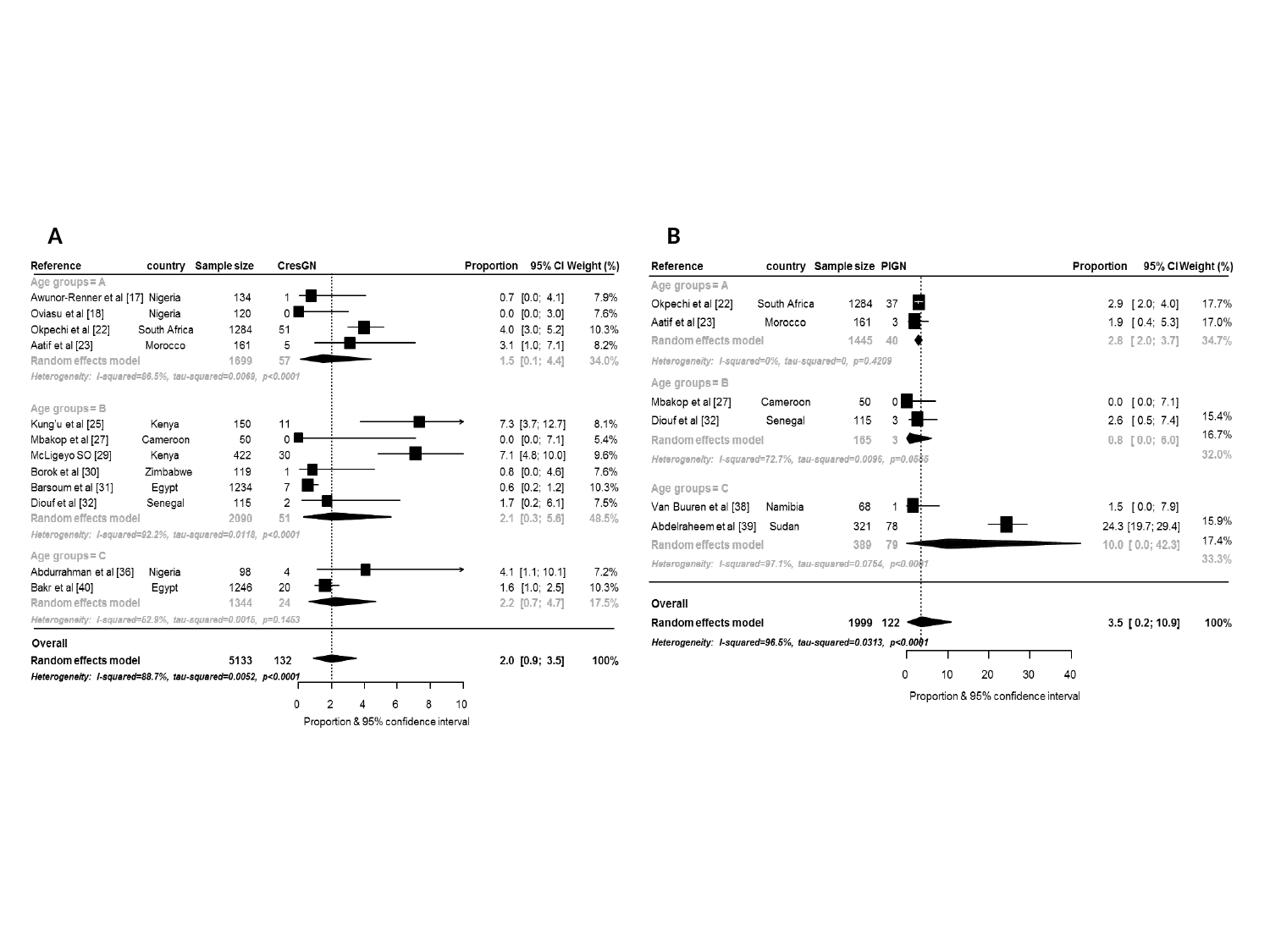

A
B

Supplement: S3 Fig — This figure shows the pooled prevalence of crescentic GN (3A) and post-infectious GN [PIGN] (3B) overall and by age group. (PPTX) [file pone.0152203.s004.pptx]

## Slide 1
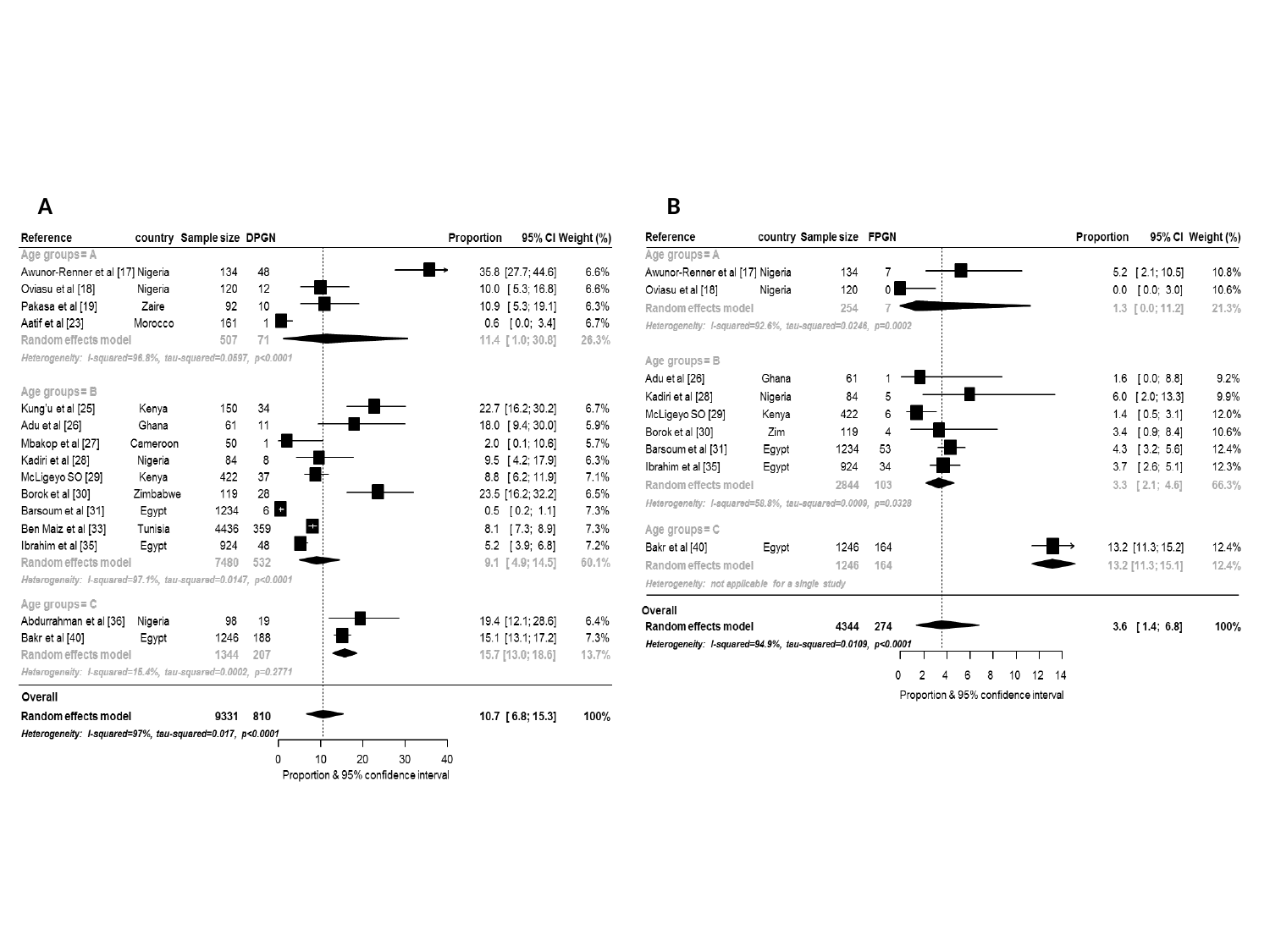

A
B

Supplement: S4 Fig — This figure shows the pooled prevalence of diffuse proliferative GN [DPGN] (4A) and focal proliferative GN [FPGN] (4B) overall and by age group. (PPTX) [file pone.0152203.s005.pptx]

## Slide 1
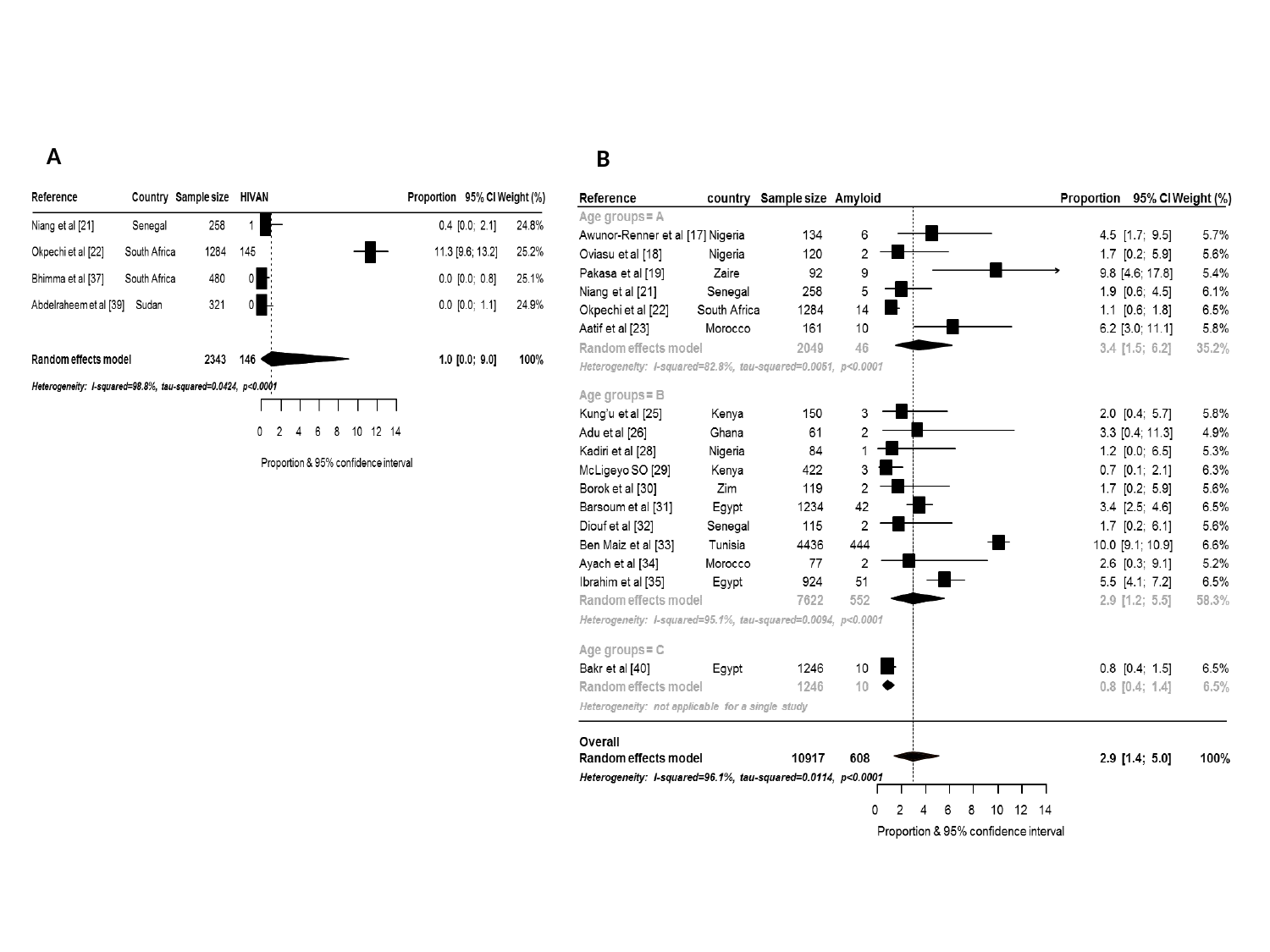

A
B

Supplement: S5 Fig — This figure shows the pooled prevalence of HIV associated nephropathy [HIVAN] (5A) and amyloidosis (5B) overall and by age group. (PPTX) [file pone.0152203.s006.pptx]
