# Supplementary material for: Epidemiology of Histologically Proven Glomerulonephritis in Africa: A Systematic Review and Meta-Analysis
Source: PLoS One. 2016 Mar 24;11(3):e0152203. doi: 10.1371/journal.pone.0152203 (PMC4806979; doi:10.1371/journal.pone.0152203)
Supplement: S1 Table — (DOCX) [file pone.0152203.s007.docx]

Supplementary Material1 1: PUBMED Search strategy

|  | **Search terms** | **Results** |
| --- | --- | --- |
| **#1a** | (glomerulonephritis"[Mesh]) OR (glomerulonephritis OR glomerulonephritides OR GN OR glomerular inflammation OR glomerulosclerosis) | 59617 |
| **#1b** | (((nephritic OR nephrotic))) | 22612 |
| **#1a and #1b in combination with the Boolean operator “or”** | ((((((((((((("glomerulonephritis"[Mesh]) OR (glomerulonephritis OR glomerulonephritides OR GN OR glomerular inflammation OR glomerulosclerosis)))) OR ((nephritic OR nephrotic))) | 73829 |
| **#2** | (renal AND biopsy) | 119848 |
| **#3** | (((((adults OR adolescent* OR pediatric* OR paediatric* OR child*)))))) | 7882907 |
| **#4** | ((((("Africa"[MeSH] OR Africa*[tw] OR Algeria[tw] OR Angola[tw] OR Benin[tw] OR Botswana[tw] OR "Burkina Faso"[tw] OR Burundi[tw] OR Cameroon[tw] OR "Canary Islands"[tw] OR "Cape Verde"[tw] OR "Central African Republic"[tw] OR Chad[tw] OR Comoros[tw] OR Congo[tw] OR "Democratic Republic of Congo"[tw] OR Djibouti[tw] OR Egypt[tw] OR "Equatorial Guinea"[tw] OR Eritrea[tw] OR Ethiopia[tw] OR Gabon[tw] OR Gambia[tw] OR Ghana[tw] OR Guinea[tw] OR "Guinea Bissau"[tw] OR "Ivory Coast"[tw] OR "Cote d'Ivoire"[tw] OR Jamahiriya[tw] OR Jamahiryia[tw] OR Kenya[tw] OR Lesotho[tw] OR Liberia[tw] OR Libya[tw] OR Libia[tw] OR Madagascar[tw] OR Malawi[tw] OR Mali[tw] OR Mauritania[tw] OR Mauritius[tw] OR Mayote[tw] OR Morocco[tw] OR Mozambique[tw] OR Mocambique[tw] OR Namibia[tw] OR Niger[tw] OR Nigeria[tw] OR Principe[tw] OR Reunion[tw] OR Rwanda[tw] OR "Sao Tome"[tw] OR Senegal[tw] OR Seychelles[tw] OR "Sierra Leone"[tw] OR Somalia[tw] OR "South Africa"[tw] OR "St Helena"[tw] OR Sudan[tw] OR Swaziland[tw] OR Tanzania[tw] OR Togo[tw] OR Tunisia[tw] OR Uganda[tw] OR "Western Sahara"[tw] OR Zaire[tw] OR Zambia[tw] OR Zimbabwe[tw] OR "Central Africa"[tw] OR "Central African"[tw] OR "West Africa"[tw] OR "West African"[tw] OR "Western Africa"[tw] OR "Western African"[tw] OR "East Africa"[tw] OR "East African"[tw] OR "Eastern Africa"[tw] OR "Eastern African"[tw] OR "North Africa"[tw] OR "North African"[tw] OR "Northern Africa"[tw] OR "Northern African"[tw] OR "South African"[tw] OR "Southern Africa"[tw] OR "Southern African"[tw] OR "sub Saharan Africa"[tw] OR "sub Saharan African"[tw] OR "subSaharan Africa"[tw] OR "subSaharan African"[tw]) NOT ("guinea pig"[tw] OR "guinea pigs"[tw] OR "aspergillus niger"[tw]))))) | 372243 |
| **#1a - #4 combined with the Boolean operator “AND”** | ((((((((((((((((("glomerulonephritis"[Mesh]) OR (glomerulonephritis OR glomerulonephritides OR GN OR glomerular inflammation OR glomerulosclerosis)))) OR ((nephritic OR nephrotic))))) AND ((((((renal AND biopsy)))))))) AND ((((((adults OR adolescent* OR pediatric* OR paediatric* OR child*)))))))) AND ((((((((("Africa"[MeSH] OR Africa*[tw] OR Algeria[tw] OR Angola[tw] OR Benin[tw] OR Botswana[tw] OR "Burkina Faso"[tw] OR Burundi[tw] OR Cameroon[tw] OR "Canary Islands"[tw] OR "Cape Verde"[tw] OR "Central African Republic"[tw] OR Chad[tw] OR Comoros[tw] OR Congo[tw] OR "Democratic Republic of Congo"[tw] OR Djibouti[tw] OR Egypt[tw] OR "Equatorial Guinea"[tw] OR Eritrea[tw] OR Ethiopia[tw] OR Gabon[tw] OR Gambia[tw] OR Ghana[tw] OR Guinea[tw] OR "Guinea Bissau"[tw] OR "Ivory Coast"[tw] OR "Cote d'Ivoire"[tw] OR Jamahiriya[tw] OR Jamahiryia[tw] OR Kenya[tw] OR Lesotho[tw] OR Liberia[tw] OR Libya[tw] OR Libia[tw] OR Madagascar[tw] OR Malawi[tw] OR Mali[tw] OR Mauritania[tw] OR Mauritius[tw] OR Mayote[tw] OR Morocco[tw] OR Mozambique[tw] OR Mocambique[tw] OR Namibia[tw] OR Niger[tw] OR Nigeria[tw] OR Principe[tw] OR Reunion[tw] OR Rwanda[tw] OR "Sao Tome"[tw] OR Senegal[tw] OR Seychelles[tw] OR "Sierra Leone"[tw] OR Somalia[tw] OR "South Africa"[tw] OR "St Helena"[tw] OR Sudan[tw] OR Swaziland[tw] OR Tanzania[tw] OR Togo[tw] OR Tunisia[tw] OR Uganda[tw] OR "Western Sahara"[tw] OR Zaire[tw] OR Zambia[tw] OR Zimbabwe[tw] OR "Central Africa"[tw] OR "Central African"[tw] OR "West Africa"[tw] OR "West African"[tw] OR "Western Africa"[tw] OR "Western African"[tw] OR "East Africa"[tw] OR "East African"[tw] OR "Eastern Africa"[tw] OR "Eastern African"[tw] OR "North Africa"[tw] OR "North African"[tw] OR "Northern Africa"[tw] OR "Northern African"[tw] OR "South African"[tw] OR "Southern Africa"[tw] OR "Southern African"[tw] OR "sub Saharan Africa"[tw] OR "sub Saharan African"[tw] OR "subSaharan Africa"[tw] OR "subSaharan African"[tw]) NOT ("guinea pig"[tw] OR "guinea pigs"[tw] OR "aspergillus niger"[tw]))))))))))) | 422 |
| **#1b-#4** | Filters: Publication date from 1980/01/01 to 2014/12/31 | 403 |
